# Face detection mechanisms: Nature *vs.* nurture

**DOI:** 10.3389/fnins.2024.1404174

**Published:** 2024-05-15

**Authors:** Dmitry Kobylkov, Giorgio Vallortigara

**Affiliations:** Centre for Mind/Brain Science, CIMeC, University of Trento, Rovereto, Italy

**Keywords:** face perception, innate, brain, neuron, stimuli

## Abstract

For many animals, faces are a vitally important visual stimulus. Hence, it is not surprising that face perception has become a very popular research topic in neuroscience, with *ca.* 2000 papers published every year. As a result, significant progress has been made in understanding the intricate mechanisms underlying this phenomenon. However, the ontogeny of face perception, in particular the role of innate predispositions, remains largely unexplored at the neural level. Several influential studies in monkeys have suggested that seeing faces is necessary for the development of the face-selective brain domains. At the same time, behavioural experiments with newborn human babies and newly-hatched domestic chicks demonstrate that a spontaneous preference towards faces emerges early in life without pre-existing experience. Moreover, we were recently able to record face-selective neural responses in the brain of young, face-naïve chicks, thus demonstrating the existence of an innate face detection mechanism. In this review, we discuss these seemingly contradictory results and propose potential experimental approaches to resolve some of the open questions.

## 1 Introduction

Although face perception is usually discussed in a social context, its functions are much more versatile. First of all, a face signals a presence of another animal, which might be a social companion (Leopold and Rhodes, 2010) or a predator (Karplus and Algom, 1981; Beránková et al., 2014). Secondly, faces play a crucial role in conveying emotional status of the animal. The social aspect of face perception is particularly prominent in humans (Ekman, 1993) and non-human primates (reviewed by Burrows, 2008) with highly developed facial muscles. However, other species such as sheep, dogs, and horses are also able to perceive emotional status through facial expressions (reviewed by Ferretti and Papaleo, 2019). Finally, facial features can provide a basis for much more specific categorization of conspecifics: identification of family members, mating partners, or single individuals (Leopold and Rhodes, 2010).

The complex process of face perception can be roughly divided into two phases: face detection and face discrimination. Face detection relies on the fact that faces are composed of features in a specific triangular arrangement (first-order relations) with eyes placed above the mouth. Conversely, a much more specific face discrimination is based on perception and learning of fine details of an individual’s face (second-order relations). Broadly speaking, discrimination does not necessarily mean unique identification of an individual (individual face recognition), and can refer to a more general categorization of, e.g., mating partners or family members. Thus, face detection serves as a broad filter which aims to rapidly drive attention to a generalized face configuration, while face discrimination process targets fine details of a particular face. Therefore, it has been suggested that the underlying neural mechanisms of face discrimination could be different from those of face detection (Johnson, 2005; Tsao and Livingstone, 2008).

The development of such a complex cognitive function as face perception is largely affected by learning and experience. At the same time, the oversimplified view on the brain of newborn animals as a *tabula rasa* is also far from reality (Vallortigara, 2021). However, the exact role of innate predispositions in the ontogeny of face perception remains a highly debated topic (Arcaro et al., 2019). Here we aim to overview the current state of this controversy and outline some of the key open questions. We will focus mainly on the mechanisms of face detection, since it represents a primary stage of face perception found already in very young animals and, hence, more likely to be influenced by innate factors. Specifically, we discuss the nature of innate face-detection mechanism: its properties and experimental challenges associated with studying this mechanism at the neural level.

## 2 Behavioural perspective

**Figure 1:**
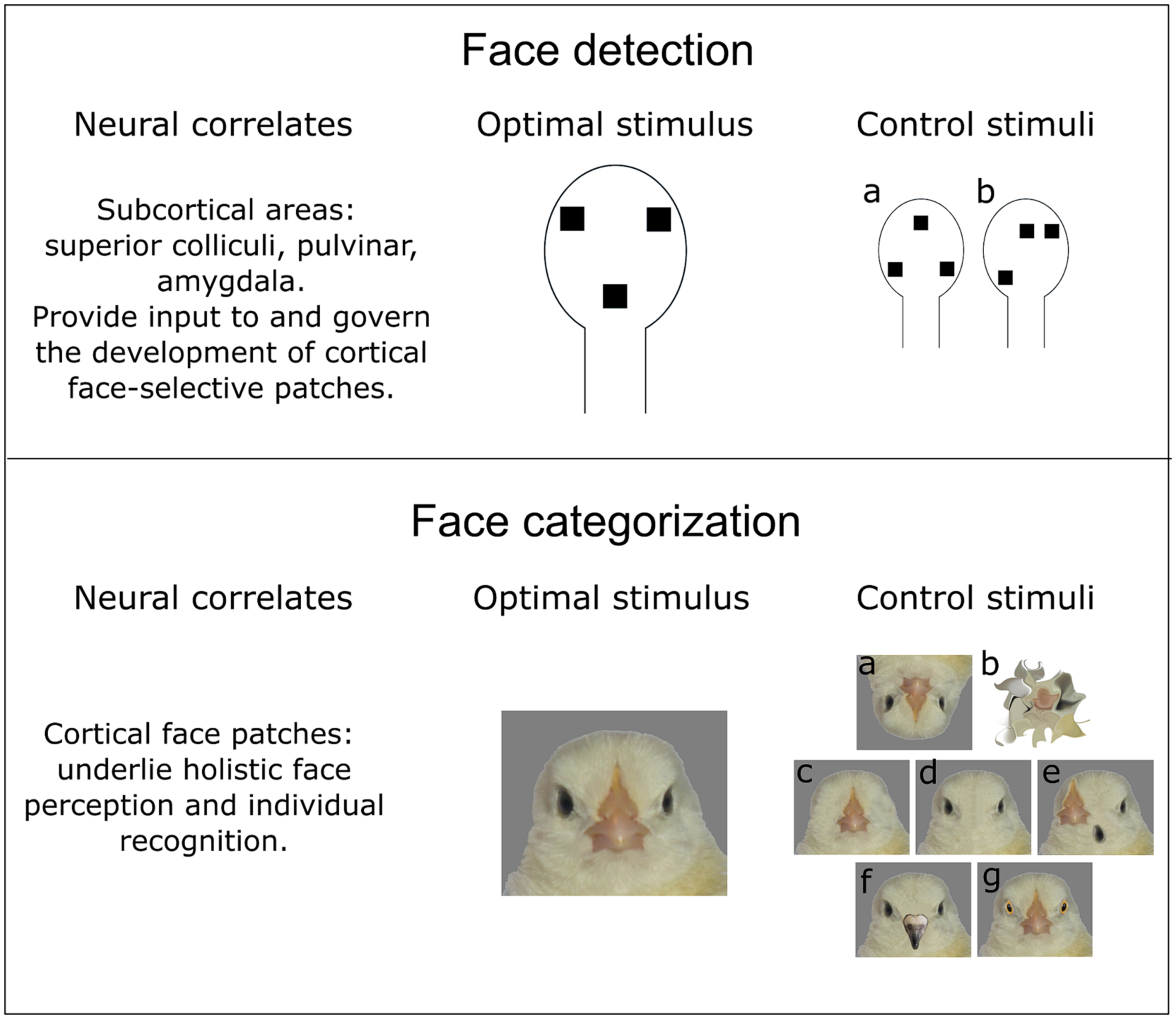
Face detection and face categorization seem to rely on different neural mechanisms. However, both processes happen simultaneously, which makes it difficult to disentangle them in the experiment. (upper part) Face detection might be an evolutionary ancestral trait of vertebrates, which relies on subcortical processing of a face-like pattern. Hence, stimuli with a schematic representation of facial features (two eyes and a mouth/beak) elicit a behavioural response in newborn animals. Two essential control stimuli could be used to describe properties of a face detector: (a) an inverted face-like pattern virtually identical to the face-like pattern in terms of spatial frequency and luminosity; (b) an asymmetric configuration to reveal a potential effect of symmetry and topheavyness (see main text) on the face detection. (lower part) In contrast to face detection, face categorization is less likely to have a common evolutionary origin among distantly-related species (such as mammals and birds). Therefore, the optimal face stimulus should be experimentally determined for each species. The control stimuli to describe the properties of a “cortical” face categorization mechanism should involve various modifications. (a) Inverted faces should affect holistic processing of facial features or face detection mechanism. (b) Scrambled faces that should preserve low-level visual characteristics (spatial frequency, luminosity) and simultaneously be semantically meaningless [e.g., through diffeomorphic transformation (Stojanoski and Cusack, 2014)]. (c, d) Faces without single facial elements or (e) with shuffled elements might reveal the feature-selectivity of neural responses. Importantly, hiding single facial elements behind the bars of a background colour is suboptimal, since it might also affect the perception of a head outline, as well as change overall spatial frequency, texture, and luminosity of a stimulus. (f, g) Chimeric faces consisting of facial elements of different individuals/species (in this case domestic chicken and pigeon) aim to investigate the selectivity of face categorization mechanism. In this case, the face detection mechanism should not be affected, since the face-like configuration is preserved.

Behavioural studies have shown that human newborns preferentially look at the face-like pattern, comprised of three dark features representing the eyes placed above the mouth (Johnson et al., 1991; Valenza et al., 1996; Figure 1). The behavioural bias towards faces has been reported already a few minutes after birth (Goren et al., 1975) or even in the uterus (Reid et al., 2017). Moreover, similar to human babies, newly hatched domestic chicks (Rosa-Salva et al., 2010, 2011) and tortoises (Versace et al., 2020) that have never seen faces before also show a spontaneous face preference. Therefore, face detection does not seem to fully rely on the early-life experience with faces and must instead derive from an innate neural mechanism.

The schematic face-like pattern remains a salient visual stimulus also in adulthood (Stein et al., 2011). This results in the phenomenon of pareidolia, when humans assign faces to inanimate objects like clouds or the Moon (Pavlova et al., 2020; Rolf et al., 2020). Rhesus monkeys are also attracted by illusory faces (Taubert et al., 2017), but at the same time they do not seem to explicitly recognize illusory faces as real faces, while humans do (Flessert et al., 2023). Apart from the response to the schematic face-like pattern and illusory faces, first-order relational properties of the face strongly influence recognition of real faces. When participants are presented with inverted faces (Taubert et al., 2011) or with faces, where the lower half belongs to another individual (Laguesse and Rossion, 2013), it impairs the face recognition. This shows that face perception in adults relies on holistic processing of the face, i.e., on first-order relations (Rossion, 2009).

The obvious similarity between holistic processing of faces observed in adults and innate face detection in newborns might suggest that the innate mechanism is simply preserved in adults. There is, however, no direct evidence that these two behaviourally similar phenomena are based on the same neural mechanism. Morton and Johnson (1991) suggested that the innate face detection mechanism (dubbed as CONSPEC) is present only during the earliest stage of life. It is then substituted by a separate CONLEARN mechanism, which is nonspecialized at the beginning and becomes tuned to faces only through experience. This hypothesis is based on experiments showing that the initial behavioural bias of newborns towards the static schematic face-like pattern slowly disappears during the first months of life (Maurer and Barrera, 1981; Johnson et al., 1991). Instead, 5-month-old infants become more attracted by moving faces with more fine details (Johnson et al., 1992).

## 3 Face detection in the adult brain

In adult humans and non-human primates, the neural mechanism of face perception has been extensively studied over the last decades, starting from seminal works on monkeys, which described neurons in the superior temporal sulcus specifically responding to faces (Bruce et al., 1981; Perrett et al., 1982). Later, fMRI studies have revealed a whole network of cortical areas that are involved in face perception. Regions with the strongest selectivity towards faces (face patches) have been identified in the inferotemporal cortex (IT) of monkeys (Tsao et al., 2003) and in the inferior occipital gyri, the lateral fusiform gyrus, and the superior temporal sulcus of humans gyrus (Kanwisher et al., 1997; Haxby et al., 1999; Ishai et al., 1999). According to the prevailing hypothesis, cortical face-selective areas in the brain of monkeys and humans form a functional hierarchy, which provides a network for individual face recognition (Freiwald and Tsao, 2010; Chang and Tsao, 2017; for review see Haxby et al., 2000; Duchaine and Yovel, 2015; Hesse and Tsao, 2020).

In contrast to individual face recognition in the cortex, rapid process of face detection might happen already at the subcortical level, as has been suggested by psychophysical experiments (Tomalski et al., 2009; Crouzet et al., 2010, but see Rossion et al., 2011). In line with this idea, at least three subcortical regions have been shown to respond to faces: the pulvinar, the superior colliculi, and the amygdala. Nguyen et al. (2013) reported the pulvinar neurons in monkeys that showed a stronger response to faces and face-like stimuli (including schematic eye-like patterns) than to simple geometric shapes. This region seems to be also involved in perception of emotional face expressions (Maior et al., 2010; Troiani and Schultz, 2013). Similarly, neurons in the superior colliculi of monkeys have been found to respond more strongly to upright and inverted face-like stimuli, than to scrambled stimuli (Le et al., 2020). Both in the pulvinar and in the superior colliculi, neural response to face-like stimuli was characterized by short latencies, which indicate that it was not modified by top-down cortical processing. However, neural responses in these subcortical regions did not show any difference between upright and inverted face-like stimuli, thus it might be premature to conclude that these responses are actually face-specific. In contrast to the pulvinar and to the superior colliculi, the role of amygdala in face perception has been described in a more detail. Single-cell recordings in human and monkey amygdala have revealed neurons preferentially tuned to facial features (Leonard et al., 1985; Gothard et al., 2007; Cao et al., 2021). Moreover, neurons in the amygdala, apparently, encode social traits of a face (Cao et al., 2021) and facial expressions (Hoffman et al., 2007; Inagaki et al., 2023).

## 4 Face detection in the newborn brain

While in the brain of adult humans and non-human primates the neural mechanism of face detection is relatively well described, it remains unclear how it corresponds to the innate face detection observed in newborns. It has been generally assumed that cortical structures continue to develop slowly after birth (Grill-Spector et al., 2008) and, thus, cannot support innate face perception. Accordingly, Livingstone et al. (2017) observed only a broad unselective response to faces in the fMRI of 1-month-old monkeys. However, other experimental evidence from humans has challenged this view (Kosakowski et al., 2022). Apparently, already during first months of life the face perception mechanism in infants is largely comparable to those in the adult brain. An fMRI study on 2-9-months-old human infants has shown face selectivity in multiple cortical regions (Kosakowski et al., 2022). Additionally, in EEG studies, face-selective responses in human infants starting from three months show activation peaks of long latencies, characteristic of cortical responses (Peykarjou and Hoehl, 2013). Moreover, while the evidence from infants might be attributed to a fast cortical specialization due to extensive face-exposure, Buiatti et al. (2019) revealed that the EEG response selective to the face-like pattern can be observed already in 1-4-days-old newborn human babies. By reconstructing the source of the EEG signal, these authors have concluded that even in newborns the face-selective responses stem (in part) from cortical areas.

However, EEG data allow only an approximate prediction of the source of the underlying neural activity. On the other hand, methods with a better spatial resolution such as fMRI and single-cell recordings cannot be implemented on newborn humans for ethical and technical reasons. In an attempt to overcome this problem, Arcaro et al. (2017) performed fMRI on juvenile (but not newborn) monkeys that had been face-deprived from birth. They have shown that development of the face patch system requires exposure to faces and, thus, is not innate. This result, however, does not necessarily refute the existence of an innate face detection mechanism, which might reside in subcortical structures rather than in the cortex. fMRI might be also not sensitive enough to detect face-selective neurons in the brain of newborns, if they are sparsely distributed and have not yet formed the face patch. Furthermore, if the main role of the innate face preference is to attract animal’s attention towards social stimuli during a sensitive period after birth and to govern the development of cortical face domains, then a long-lasting face-deprivation might lead to an abnormal cortical development and behaviour (Benetti et al., 2017). Interestingly, Arcaro et al. (2017) also have noticed that control monkeys were attracted to faces even before the emergence of the face domains in the brain, while the face-deprived monkeys were not interested in faces at all. While authors see it as evidence against an innate nature of face-looking behaviour, one could also interpret this result as indirect evidence for a separate innate mechanism of face detection that precedes the development of cortical face-selective areas.

To avoid long periods of face deprivation and study innate face detection mechanisms, an alternative solution would be to use precocial species such as domestic chicks. These animals show spontaneous behavioural bias towards faces soon after hatching. Recently, we were able to record neural activity in a higher associative brain area of chicks that might underlie this innate face preference (Kobylkov et al., 2024). Neurons in the caudolateral nidopallium showed a strong selective response to the face-like schematic pattern. These neurons were tuned to a canonical face-like configuration of facial features and responded much less to inverted or asymmetric stimuli. However, it is important to note that in adult chickens the mechanism of individual recognition is still unknown (but see Town, 2011). Therefore, the potential role of these innate face detectors in the development of any mammal-like cortical face domains remains elusive.

## 5 Models of innate face detection

The idea that the neural networks in the brain are preconfigured in some way already at birth has become largely accepted in neuroscience (e.g., Dragoi, 2024; Powell et al., 2024). However, the main open question and also the main arguing point is related to underlying principles of this innate brain configurations and what is the level of categorization this innate system is able to achieve before being shaped by experience. In the case of face detection, we should answer the question of whether newborns are actually able to detect “facedness,” i.e., the first-order configuration of facial features, or if their response might be triggered by some lower-level physical parameters of the stimulus.

To explain the innate preference of newborn animals towards faces, Morton and Johnson (1991) proposed the existence of an innate mechanism capable of detecting the first-order face configuration. The innate face detector would be, thus, sensitive to the high-contrast blobs representing facial features in a canonical triangular configuration. The main counterargument against this hypothesis is the complexity of the face-like pattern: the existence of a putative face detector that would be able to selectively respond only to a specific number of elements in a well-defined configuration seems too sophisticated for an innate structure. However, innate visual object recognition *per se* is not rare, and we actually know the neural mechanisms for at least some of these behaviours. For example, zebrafish larvae have a dedicated neural pathway that underlies their innate response towards a small moving prey (Semmelhack et al., 2014). Although recognition of the face-like pattern is a more challenging visual task, it is still plausible that the neural basis of this behaviour is present from birth. Face detectors in the brain of face-naïve domestic chicks seem to fulfil these criteria, showing that it is possible to have an innate mechanism selective for a face-like configuration.

Alternatively, Arcaro and Livingstone (2021) have proposed that domain-specific categorization of objects including faces does not require any innate predispositions and develops with experience. A topographic map based on retinotopic organization is present in the brain of monkeys from birth (Arcaro and Livingstone, 2017). This retinotopically-based organization could serve as a scaffold for development of object-selective domains in the cortex of primates, where faces are strongly represented in the central foveal visual field (Kamps et al., 2020). According to the “bottom-up” model of Arcaro and Livingstone (2021), the selective properties of the proto-map in this central field is what dictates the specialization of corresponding cortical domains. In other words, the proto-architecture was not evolved to be face-selective; instead, the low-level properties of a face (e.g., its curvature and eccentricity) are happen to be the best stimuli for the corresponding central field neurons. At the same time, it remains unclear why do central field areas have exactly these properties if not because of the evolutionary adaptation to perceive specific visual stimuli that are highly important for the survival (like faces).

Similar to many other sensory hypotheses, the bottom-up model suggests that innate face detection could be explained by characteristics of the sensory system rather than properties of the stimulus. Among low-level characteristics that have been suggested to underlie the innate face preference is low spatial frequency (reviewed by Simion et al., 2007) and topheavyness [more elements in the upper part of the stimulus, (Simion et al., 2002; Turati et al., 2002)]. However, none of these properties alone seem to fully explain the face-preference observed in newborn animals. Animals differentiate between upright and inverted face-like pattern with identical spatial frequency; there is also no general bias towards top-heavy stimuli either in chicks (Rosa-Salva et al., 2010; Kobylkov et al., 2024) nor in human newborns (as revealed by EEG recordings, Buiatti et al., 2019). In addition, there are multiple other visual properties that have been shown to affect face detection in newborns, such as contrast polarity (Farroni et al., 2005), vertical symmetry of face elements (Buiatti et al., 2019), and the shape of the head outline (Macchi Cassia et al., 2008). Altogether, it appears that for a sensory hypothesis to fully explain the behavioural preference towards faces it should include not one, but many sensory filters for multiple parameters. This, in turn, would suggest that innate face preference cannot be fully reduced to a by-product of sensory limitations or to one particular low-level physical variable.

How likely is it that the innate face preference is driven by the same cortical domains found in adults? The only way to directly answer this question is by recording neural activity in newborn animals. To date, both the EEG study in human neonates (Buiatti et al., 2019) and single-cell recordings in domestic chicks (Kobylkov et al., 2024) have shown face-selective responses in cortical structures. This, however, should not be seen as ultimate proof of fully-functioning face-selective cortical domains in newborns. Instead, this neural activity might reflect the feedforward propagation of signals from subcortical areas (Behrmann and Avidan, 2022). In case of face-selective neurons in the endbrain of chicks, this idea is indirectly supported by results of a time-resolved analysis. It revealed that a face-like stimulus can be decoded from raw neural population response soon after the stimulus onset, suggesting that the processing of faces could happen at the earlier subcortical stage. The exact location of these putative subcortical face detectors remains unknown. However, the amygdala appears to be a promising candidate region, since it was shown to be selectively activated by social stimuli in newly-hatched chicks (Mayer et al., 2017, 2019).

## 6 Discussion

Although we are far from a comprehensive understanding on how the face detection mechanism develops in the brain, we can summarize what appears to be known so far and what remains an open question.

1. Spontaneous behavioural bias towards faces observed in newborn animals is most likely driven by an innate neural mechanism, which enables detection of a face-like configuration. However, it remains unclear, whether face detection mechanism in newborns is based on the same neural correlates as in the adult brain. Moreover, the involvement of cortical areas of neonates into face processing might be species-specific.
2. The innate face detection mechanism could be based on the retinotopic proto-architecture of the brain, which is present at birth. In this case, brain areas receiving information from the central foveal visual field might preferentially respond to face-like stimuli. At the same time, so far this hypothesis has not been tested on newborn animals responding to schematic face-like stimuli.
3. We believe that a potential source of discrepancy between results on face perception in neonates stems from the fact that they are obtained in different species (humans, monkeys, and birds), by different methods of neural recording (EEG, fMRI, and extracellular recordings), and using different visual stimuli (naturalistic faces vs. face-like patterns). Recording techniques often cannot be significantly optimized due to technical limitations (spatial and temporal resolution (Logothetis, 2008), species-specific constraints). Therefore, we would like to outline two other potentially important sources of discrepancy and the ways to deal with it.

### 6.1 Importance of stimuli

The ultimate goal of neuroscience is to understand naturalistic behaviour of animals. At the same time, the experimental procedure is inevitably far from a realistic scenario: in nature animals never experience a random fast presentation of static faces (without a body) on a flat screen. Therefore, in an attempt to dissect the complex face perception phenomenon, it might be particularly useful to combine naturalistic face images with schematic face-like patterns in experiments. The latter serves as a “supernormal” stimulus (Tinbergen, 1953) allowing to selectively trigger and differentiate between face detection and face recognition mechanisms. Instead, static images of naturalistic faces might vary in illumination and, hence, in contrast of facial features. Newborn animals with low visual acuity, such as primates, might be less attentive to such stimuli, although they appear more naturalistic to an adult’s eye. Hence, we believe that the combination of both naturalistic and schematic face-like stimuli would be advantageous for understanding the face perception mechanisms.

Another important aspect relates to the use of “scrambled faces” to control for face-selectivity of neural responses. There is a high discrepancy between studies in what they call a “scrambled” stimulus: from face-like patterns with altered vertical symmetry (Buiatti et al., 2019) to face images modified based on the parametric texture model (Livingstone et al., 2017) or diffeomorphic transformation (Stojanoski and Cusack, 2014). In all cases it would be important to estimate the perceived “facedness” of such stimuli by animals (but see Bardon et al., 2022), because even an automatic scrambling approach might accidentally produce an illusory face-like pattern. Conversely, since behavioural tests with newborn animals are often challenging, one might consider to estimate the “facedness” of stimuli by a neural network that has been trained on face detection.

Additionally, even when the experimental design involves a variety of faces and face-like stimuli, as well as controls for low-level properties, claims of exclusive face-selectivity should be made with caution. In a recent study, Vinken et al. (2023) have demonstrated that neurons in the inferotemporal cortex of adult monkeys have graded response profiles towards non-face objects. Authors suggest that face-selectivity cannot be explained by face-specific features only and advocate for a larger variety of tested stimuli (Figure 1).

### 6.2 Importance of comparative approach

Without any doubts, primates are masters of face perception, and research on primates has brought ground-breaking discoveries in this field. Nevertheless, there are several reasons advocating for diversity of model systems in neuroscience (Laurent, 2020), in particular when studying ontogeny of neural mechanisms. First of all, any attempt to study innate properties of the brain in an altricial species like humans or monkeys is inevitably associated with certain limitations: newborn animals do not show stable behaviour, the sensory system is underdeveloped, and it is difficult to control for the early-life exposure. Conversely, precocial animals that are born on a more advanced developmental stage can be tested when truly naïve to faces.

Moreover, while some experimental results from humans, monkeys, and domestic chicks might appear controversial, it is important to take into account possible species-specific differences. For example, newborn primates have much lower visual acuity (Boothe et al., 1985) than newly-hatched chicks (Jarvis et al., 2009), which might result in different properties of an innate face detector. On the other hand, it also worth noting that even among primates, individual face recognition skills are not as ubiquitous as they might appear. Rhesus monkeys, for example, are poor at discriminating individual faces of conspecifics and do not show a systematic inversion effect (Rossion and Taubert, 2019; Griffin, 2020). Therefore, mechanisms of face recognition in non-human species should not necessarily be identical or even similar to those in the human brain.

## Author contributions

DK: Conceptualization, Writing – original draft, Writing – review & editing. GV: Funding acquisition, Project administration, Writing – original draft, Writing – review & editing.
